# Carvedilol dihydrogen phosphate propan-2-ol solvate from powder diffraction data

**DOI:** 10.1107/S1600536810005349

**Published:** 2010-02-13

**Authors:** Vladimir V. Chernyshev, Sergei Yu. Kukushkin, Yurii A. Velikodny

**Affiliations:** a Department of Chemistry, Moscow State University, 119991 Moscow, Russian Federation; b BION Ltd, 109 km., Kiev Highway, Obninsk 249032, Kaluga Region, Russian Federation

## Abstract

In the cation of the title compound, C_24_H_27_N_2_O_4_^+^·H_2_PO_4_^−^·C_3_H_8_O [systematic name: 3-(9*H*-carbazol-4-yl­oxy)-2-hydr­oxy-*N*-[2-(2-methoxy­phen­oxy)eth­yl]propan-1-aminium dihydro­gen phosphate propan-2-ol solvate], the mean planes of the tricyclic carbazole system and the benzene ring form a dihedral angle of 42.00 (16)°. In the crystal structure, classical inter­molecular O—H⋯O and N—H⋯O hydrogen bonds link the cations, anions and solvent mol­ecules into layers parallel to the *ac* plane.

## Related literature

For details of the synthesis, see: Brook *et al.* (2005 ▶). For the indexing algorithm, see: Werner *et al.* (1985 ▶). For the crystal structures of carvedilol as a free base and a cation, see: Chen *et al.* (1998 ▶); Yathirajan *et al.* (2007 ▶); Chernyshev *et al.* (2009 ▶). 
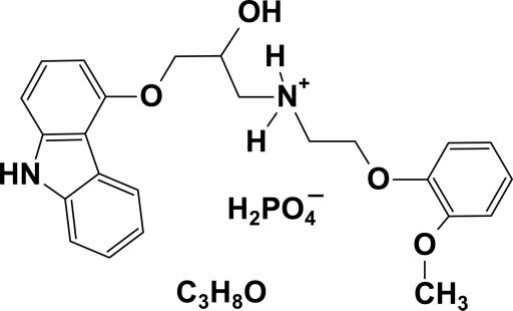


## Experimental

### 

#### Crystal data

C_24_H_27_N_2_O_4_^+^·H_2_PO_4_^−^·C_3_H_8_O
*M* *_r_* = 564.56
Triclinic, 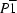

*a* = 11.5516 (11) Å
*b* = 16.6523 (19) Å
*c* = 7.8643 (8) Å
α = 95.404 (15)°
β = 94.635 (16)°
γ = 71.247 (14)°
*V* = 1424.1 (3) Å^3^
*Z* = 2
Cu *K*α_1_ radiation, λ = 1.54059 Å
μ = 1.32 mm^−1^
*T* = 295 K
flat_sheet, 15 × 1 mm

#### Data collection

G670 Guinier camera imaging plate diffractometer
Specimen mounting: thin layer in the specimen holder of the camera
Data collection mode: transmission
Scan method: continuous
2θ_min_ = 3.50°, 2θ_max_ = 85.00°, 2θ_step_ = 0.01°

#### Refinement

*R*_p_ = 0.020
*R*_wp_ = 0.026
*R*_exp_ = 0.012
*R*_Bragg_ = 0.051
χ^2^ = 4.516
8151 data points
183 parameters
134 restraints
H-atom parameters constrained

### 

Data collection: *G670 Imaging Plate Guinier Camera Software* (Huber, 2002 ▶); cell refinement: *MRIA* (Zlokazov & Chernyshev, 1992 ▶); data reduction: *G670 Imaging Plate Guinier Camera Software*; method used to solve structure: simulated annealing (Zhukov *et al.*, 2001 ▶); program(s) used to refine structure: *MRIA*; molecular graphics: *PLATON* (Spek, 2009 ▶); software used to prepare material for publication: *MRIA* and *SHELXL97* (Sheldrick, 2008 ▶).

## Supplementary Material

Crystal structure: contains datablocks I, global. DOI: 10.1107/S1600536810005349/ya2118sup1.cif

Rietveld powder data: contains datablocks I. DOI: 10.1107/S1600536810005349/ya2118Isup2.rtv

Additional supplementary materials:  crystallographic information; 3D view; checkCIF report

## Figures and Tables

**Table 1:** Hydrogen-bond geometry (Å, °)

*D*—H⋯*A*	*D*—H	H⋯*A*	*D*⋯*A*	*D*—H⋯*A*
O18—H18⋯O34	0.82	1.89	2.681 (10)	165
N19—H19*A*⋯O34^i^	0.90	1.75	2.568 (10)	149
N19—H19*B*⋯O18	0.90	2.46	2.772 (11)	101
N19—H19*B*⋯O22	0.90	2.29	2.646 (10)	103
O32—H32⋯O33^ii^	0.82	1.90	2.672 (10)	156
O35—H35⋯O36^iii^	0.82	1.99	2.619 (9)	132
O36—H36⋯O33	0.82	2.00	2.590 (9)	128

Symmetry codes: (i) 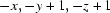; (ii) 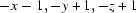; (iii) 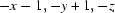.

**Fig. 1.**
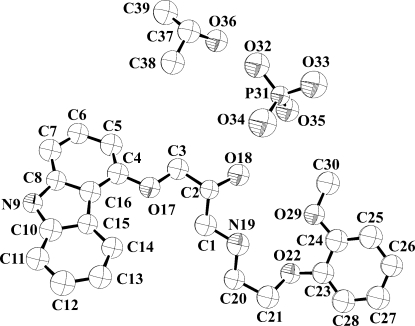
The molecular structure of (I) with the atomic numbering and 50% displacement spheres.

**Fig. 2.**
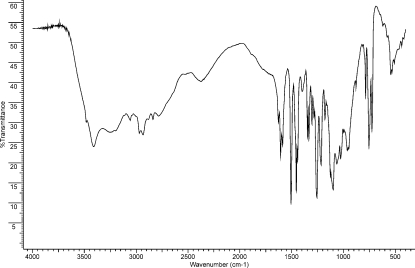
IR spectrum for (I).

**Fig. 3.**
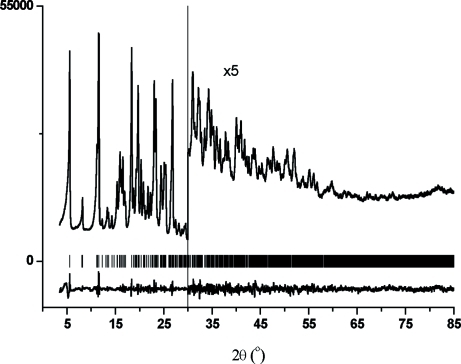
The Rietveld plot, showing the observed and difference profiles for (I). The reflection positions are shown above the difference profile.

## supplementary crystallographic information

### Comment

The crystal structures of two polymorphs of carvedilol free base (Chen *et al.*, 1998; Yathirajan *et al.*, 2007) and carvedilol dihydrogen phosphate hemihydrate Form I (Chernyshev *et al.*, 2009) were recently reported. As a contribution to structural study of carvedilol, herewith we report the crystal structure of the title compound (I) (Fig. 1).

One of two amino H atoms (H19B) is involved in intramolecular N—H···O hydrogen bonds (Table 1), which influence the cation conformation. The mean planes of the tricyclic carbazole system and the benzene ring form a dihedral angle of 42.00 (16)°. In the crystal structure, the classical intermolecular O—H···O and N—H···O hydrogen bonds (Table 1) link cations, anions and solvent molecules into layers parallel to the *ac* plane.

### Experimental

Carvedilol dihydrogen phosphate hemihydrate (*CDPH*) was synthesized in accordance with the known procedure (Brook *et al.*, 2005). Synthesis of carvedilol dihydrogen phosphate isopropanol solvate was carried out under stirring in round bottom four necked flask (0.5 L) equipped with thermometer and heated addition funnel. A hot solution (55-60°C) of *CDPH* (4 g, 8 mmol) in MeOH (70 ml) was placed in heated (60°C) addition funnel. Then this solution was added dropwise to isopropanol (250 ml) for 40 minutes at -20°C. The mixture was left to stand at -17°C for 20 h. The precipitate was filtered on suction funnel, washed with cooled (10°C) isopropanol (20 ml) and then dried under vacuum at 45°C for 17 h. Carvedilol dihydrogen phosphate isopropanol solvate (3.8 g, yield 95%) was obtained with purity of 99.8 % (measured with HPLC). IR-spectrum is shown on Fig. 2.

### Refinement

During the exposure, the specimen was spun in its plane to improve particle statistics. The triclinic unit cell dimensions were determined with the indexing program TREOR (Werner *et al.*, 1985), M_20_ = 43, using the first 30 peak positions. The structure of (I) was solved by simulated annealing procedure (Zhukov *et al.*, 2001) and refined following the methodology described in (Chernyshev *et al.*, 2009). All non-H atoms were isotropically refined. H atoms were placed in geometrically calculated positions (O—H 0.82 Å; N—H 0.86–0.90 Å; C—H 0.93–0.98 Å) and included in the refinement in riding motion approximation [U_iso_(H) = 1.2U_eq_ of the carrying atom (1.5U_eq_ for O-H and Me groups)]. The diffraction profiles and the differences between the measured and calculated profiles are shown in Fig. 3.

### Crystal data

**Table:**

C_24_H_27_N_2_O_4_^+^·H_2_PO_4_^−^·C_3_H_8_O	*Z* = 2
*M**_r_* = 564.56	*F*(000) = 600
Triclinic, *P*1	*D*_x_ = 1.317 Mg m^−^^3^
*a* = 11.5516 (11) Å	Cu *K*α_1_ radiation, λ = 1.54059 Å
*b* = 16.6523 (19) Å	µ = 1.32 mm^−^^1^
*c* = 7.8643 (8) Å	*T* = 295 K
α = 95.404 (15)°	Particle morphology: no specific habit
β = 94.635 (16)°	light grey
γ = 71.247 (14)°	flat_sheet, 15 × 1 mm
*V* = 1424.1 (3) Å^3^	Specimen preparation: Prepared at 295 K and 101 kPa

### Data collection

**Table:**

G670 Guinier camera imaging plate diffractometer	Data collection mode: transmission
Radiation source: line-focus sealed tube	Scan method: continuous
Curved Germanium (111)	2θ_min_ = 3.50°, 2θ_max_ = 85.00°, 2θ_step_ = 0.01°
Specimen mounting: thin layer in the specimen holder of the camera	

### Refinement

**Table:**

Refinement on *I*_net_	Profile function: split-type pseudo-Voigt (Toraya, 1986)
Least-squares matrix: full with fixed elements per cycle	183 parameters
*R*_p_ = 0.020	134 restraints
*R*_wp_ = 0.026	0 constraints
*R*_exp_ = 0.012	H-atom parameters constrained
*R*_Bragg_ = 0.051	Weighting scheme based on measured s.u.'s
χ^2^ = 4.516	(Δ/σ)_max_ = 0.002
8151 data points	Background function: Chebyshev polynomial up to the 5th order
Excluded region(s): none	Preferred orientation correction: March-Dollase (Dollase, 1986); direction of preferred orientation 001, texture parameter *r* = 0.978(4).

### Special details

**Table:**

Geometry. All esds (except the esd in the dihedral angle between two l.s. planes) are estimated using the full covariance matrix. The cell esds are taken into account individually in the estimation of esds in distances, angles and torsion angles; correlations between esds in cell parameters are only used when they are defined by crystal symmetry. An approximate (isotropic) treatment of cell esds is used for estimating esds involving l.s. planes.

### Fractional atomic coordinates and isotropic or equivalent isotropic displacement parameters (Å^2^)

**Table:**

	*x*	*y*	*z*	*U*_iso_*/*U*_eq_	
C1	0.1569 (9)	0.4372 (6)	0.1923 (11)	0.081 (5)*	
H1A	0.2323	0.3959	0.2322	0.097*	
H1B	0.1532	0.4318	0.0682	0.097*	
C2	0.0481 (8)	0.4175 (5)	0.2558 (11)	0.060 (4)*	
H2	0.0602	0.4105	0.3790	0.072*	
C3	0.0364 (9)	0.3355 (5)	0.1605 (11)	0.075 (5)*	
H3A	−0.0284	0.3196	0.2051	0.090*	
H3B	0.0184	0.3429	0.0393	0.090*	
C4	0.1649 (9)	0.1898 (5)	0.1249 (12)	0.074 (4)*	
C5	0.0787 (9)	0.1645 (6)	0.0178 (11)	0.082 (5)*	
H5	0.0051	0.2044	−0.0149	0.098*	
C6	0.1032 (9)	0.0783 (5)	−0.0411 (11)	0.073 (5)*	
H6	0.0436	0.0621	−0.1089	0.087*	
C7	0.2137 (9)	0.0168 (6)	−0.0007 (13)	0.081 (4)*	
H7	0.2283	−0.0400	−0.0383	0.097*	
C8	0.3021 (8)	0.0440 (6)	0.0988 (11)	0.063 (4)*	
N9	0.4179 (7)	−0.0027 (4)	0.1591 (9)	0.059 (3)*	
H9	0.4523	−0.0562	0.1356	0.071*	
C10	0.4702 (9)	0.0498 (6)	0.2632 (11)	0.078 (5)*	
C11	0.5820 (9)	0.0296 (6)	0.3584 (12)	0.077 (5)*	
H11	0.6350	−0.0259	0.3570	0.092*	
C12	0.6116 (9)	0.0955 (6)	0.4557 (12)	0.090 (5)*	
H12	0.6876	0.0842	0.5148	0.108*	
C13	0.5289 (9)	0.1788 (6)	0.4663 (12)	0.077 (4)*	
H13	0.5490	0.2209	0.5370	0.093*	
C14	0.4171 (9)	0.1988 (6)	0.3720 (11)	0.068 (4)*	
H14	0.3638	0.2543	0.3766	0.082*	
C15	0.3860 (8)	0.1341 (5)	0.2696 (11)	0.062 (4)*	
C16	0.2776 (9)	0.1293 (5)	0.1691 (12)	0.073 (5)*	
O17	0.1515 (5)	0.2726 (4)	0.1889 (7)	0.070 (3)*	
O18	−0.0596 (6)	0.4879 (4)	0.2224 (7)	0.079 (3)*	
H18	−0.1171	0.4835	0.2714	0.119*	
N19	0.1589 (7)	0.5250 (5)	0.2533 (9)	0.085 (4)*	
H19A	0.1570	0.5309	0.3680	0.102*	
H19B	0.0910	0.5632	0.2106	0.102*	
C20	0.2692 (9)	0.5444 (6)	0.2024 (12)	0.074 (4)*	
H20A	0.2722	0.5381	0.0788	0.089*	
H20B	0.3429	0.5043	0.2500	0.089*	
C21	0.2643 (9)	0.6346 (6)	0.2664 (12)	0.086 (5)*	
H21A	0.2850	0.6378	0.3883	0.103*	
H21B	0.3210	0.6529	0.2076	0.103*	
O22	0.1417 (6)	0.6860 (4)	0.2302 (7)	0.070 (3)*	
C23	0.0870 (9)	0.7546 (6)	0.3420 (12)	0.080 (5)*	
C24	−0.0359 (9)	0.7649 (6)	0.3745 (12)	0.088 (5)*	
C25	−0.1014 (9)	0.8340 (6)	0.4794 (11)	0.091 (5)*	
H25	−0.1817	0.8403	0.5024	0.109*	
C26	−0.0462 (9)	0.8941 (6)	0.5502 (12)	0.079 (4)*	
H26	−0.0902	0.9404	0.6197	0.095*	
C27	0.0740 (9)	0.8846 (6)	0.5168 (13)	0.090 (5)*	
H27	0.1095	0.9255	0.5613	0.108*	
C28	0.1419 (9)	0.8137 (6)	0.4164 (12)	0.089 (5)*	
H28	0.2236	0.8060	0.3994	0.107*	
O29	−0.0815 (6)	0.7013 (4)	0.3012 (8)	0.073 (3)*	
C30	−0.2122 (9)	0.7240 (6)	0.2683 (12)	0.093 (5)*	
H30A	−0.2328	0.6752	0.2179	0.140*	
H30B	−0.2387	0.7686	0.1913	0.140*	
H30C	−0.2521	0.7432	0.3739	0.140*	
P31	−0.3639 (3)	0.52870 (19)	0.3615 (4)	0.0600 (14)*	
O32	−0.4390 (6)	0.5878 (4)	0.4988 (9)	0.115 (3)*	
H32	−0.4775	0.5615	0.5402	0.173*	
O33	−0.4183 (6)	0.4583 (4)	0.3049 (8)	0.101 (3)*	
O34	−0.2334 (7)	0.4907 (4)	0.4313 (8)	0.127 (3)*	
O35	−0.3653 (6)	0.5787 (4)	0.2088 (8)	0.092 (3)*	
H35	−0.4098	0.5644	0.1337	0.138*	
O36	−0.4622 (6)	0.3709 (4)	0.0315 (8)	0.085 (3)*	
H36	−0.4178	0.3987	0.0706	0.128*	
C37	−0.4205 (9)	0.2878 (6)	0.0907 (12)	0.089 (5)*	
H37	−0.4595	0.2880	0.1970	0.107*	
C38	−0.2846 (9)	0.2616 (6)	0.1240 (13)	0.093 (5)*	
H38A	−0.2642	0.3007	0.2095	0.140*	
H38B	−0.2464	0.2619	0.0202	0.140*	
H38C	−0.2561	0.2054	0.1635	0.140*	
C39	−0.4550 (10)	0.2278 (6)	−0.0426 (13)	0.105 (5)*	
H39A	−0.5424	0.2455	−0.0623	0.158*	
H39B	−0.4272	0.1714	−0.0037	0.158*	
H39C	−0.4175	0.2280	−0.1471	0.158*	

### Geometric parameters (Å, °)

**Table:**

C1—N19	1.502 (12)	C20—H20A	0.97
C1—C2	1.528 (15)	C20—H20B	0.97
C1—H1A	0.97	C21—O22	1.421 (10)
C1—H1B	0.97	C21—H21A	0.97
C2—O18	1.435 (9)	C21—H21B	0.97
C2—C3	1.533 (13)	O22—C23	1.383 (10)
C2—H2	0.98	C23—C28	1.395 (15)
C3—O17	1.420 (10)	C23—C24	1.415 (15)
C3—H3A	0.97	C24—O29	1.388 (13)
C3—H3B	0.97	C24—C25	1.392 (12)
C4—O17	1.387 (11)	C25—C26	1.402 (16)
C4—C5	1.395 (14)	C25—H25	0.93
C4—C16	1.407 (12)	C26—C27	1.390 (15)
C5—C6	1.410 (13)	C26—H26	0.93
C5—H5	0.93	C27—C28	1.400 (12)
C6—C7	1.390 (12)	C27—H27	0.93
C6—H6	0.93	C28—H28	0.93
C7—C8	1.401 (14)	O29—C30	1.441 (12)
C7—H7	0.93	C30—H30A	0.96
C8—N9	1.385 (10)	C30—H30B	0.96
C8—C16	1.422 (12)	C30—H30C	0.96
N9—C10	1.387 (13)	P31—O32	1.513 (7)
N9—H9	0.86	P31—O34	1.514 (8)
C10—C11	1.398 (13)	P31—O33	1.516 (8)
C10—C15	1.427 (11)	P31—O35	1.520 (7)
C11—C12	1.395 (14)	O32—H32	0.82
C11—H11	0.93	O35—H35	0.82
C12—C13	1.408 (12)	O36—C37	1.421 (11)
C12—H12	0.93	O36—H36	0.82
C13—C14	1.394 (13)	C37—C38	1.496 (14)
C13—H13	0.93	C37—C39	1.499 (14)
C14—C15	1.406 (13)	C37—H37	0.98
C14—H14	0.93	C38—H38A	0.96
C15—C16	1.447 (14)	C38—H38B	0.96
O18—H18	0.82	C38—H38C	0.96
N19—C20	1.503 (14)	C39—H39A	0.96
N19—H19A	0.90	C39—H39B	0.96
N19—H19B	0.90	C39—H39C	0.96
C20—C21	1.523 (13)		
			
N19—C1—C2	112.3 (7)	N19—C20—C21	110.7 (7)
N19—C1—H1A	109.2	N19—C20—H20A	109.5
C2—C1—H1A	109.2	C21—C20—H20A	109.5
N19—C1—H1B	109.1	N19—C20—H20B	109.5
C2—C1—H1B	109.1	C21—C20—H20B	109.5
H1A—C1—H1B	107.9	H20A—C20—H20B	108.1
O18—C2—C1	107.6 (7)	O22—C21—C20	105.5 (8)
O18—C2—C3	110.5 (7)	O22—C21—H21A	110.6
C1—C2—C3	109.4 (7)	C20—C21—H21A	110.7
O18—C2—H2	109.7	O22—C21—H21B	110.6
C1—C2—H2	109.7	C20—C21—H21B	110.6
C3—C2—H2	109.8	H21A—C21—H21B	108.7
O17—C3—C2	105.5 (7)	C23—O22—C21	119.7 (7)
O17—C3—H3A	110.6	O22—C23—C28	125.2 (9)
C2—C3—H3A	110.6	O22—C23—C24	115.2 (9)
O17—C3—H3B	110.6	C28—C23—C24	119.6 (8)
C2—C3—H3B	110.7	O29—C24—C25	124.2 (10)
H3A—C3—H3B	108.7	O29—C24—C23	115.7 (7)
O17—C4—C5	125.1 (7)	C25—C24—C23	120.0 (10)
O17—C4—C16	115.3 (8)	C24—C25—C26	119.9 (10)
C5—C4—C16	119.6 (8)	C24—C25—H25	120.1
C4—C5—C6	120.0 (8)	C26—C25—H25	120.0
C4—C5—H5	120.0	C27—C26—C25	120.1 (8)
C6—C5—H5	120.0	C27—C26—H26	119.9
C7—C6—C5	122.1 (9)	C25—C26—H26	120.0
C7—C6—H6	119.0	C26—C27—C28	120.3 (10)
C5—C6—H6	119.0	C26—C27—H27	119.9
C6—C7—C8	117.3 (9)	C28—C27—H27	119.9
C6—C7—H7	121.4	C23—C28—C27	120.0 (10)
C8—C7—H7	121.3	C23—C28—H28	120.0
N9—C8—C7	129.6 (8)	C27—C28—H28	120.0
N9—C8—C16	108.1 (8)	C24—O29—C30	117.0 (7)
C7—C8—C16	122.0 (8)	O29—C30—H30A	109.5
C8—N9—C10	109.8 (7)	O29—C30—H30B	109.5
C8—N9—H9	125.1	H30A—C30—H30B	109.5
C10—N9—H9	125.1	O29—C30—H30C	109.5
N9—C10—C11	129.7 (8)	H30A—C30—H30C	109.5
N9—C10—C15	108.6 (8)	H30B—C30—H30C	109.5
C11—C10—C15	121.6 (9)	O32—P31—O34	109.5 (4)
C12—C11—C10	117.9 (8)	O32—P31—O33	109.8 (4)
C12—C11—H11	121.1	O34—P31—O33	109.7 (4)
C10—C11—H11	121.1	O32—P31—O35	109.2 (4)
C11—C12—C13	121.3 (9)	O34—P31—O35	109.6 (5)
C11—C12—H12	119.3	O33—P31—O35	109.1 (4)
C13—C12—H12	119.3	P31—O32—H32	107
C14—C13—C12	120.7 (9)	P31—O35—H35	106
C14—C13—H13	119.6	C37—O36—H36	111
C12—C13—H13	119.7	O36—C37—C38	109.2 (9)
C13—C14—C15	119.1 (8)	O36—C37—C39	108.8 (8)
C13—C14—H14	120.4	C38—C37—C39	110.6 (8)
C15—C14—H14	120.4	O36—C37—H37	109.4
C14—C15—C10	119.2 (8)	C38—C37—H37	109.4
C14—C15—C16	134.5 (7)	C39—C37—H37	109.4
C10—C15—C16	106.1 (8)	C37—C38—H38A	109.5
C4—C16—C8	118.8 (9)	C37—C38—H38B	109.5
C4—C16—C15	133.7 (8)	H38A—C38—H38B	109.5
C8—C16—C15	107.3 (7)	C37—C38—H38C	109.5
C4—O17—C3	118.1 (7)	H38A—C38—H38C	109.4
C2—O18—H18	110	H38B—C38—H38C	109.5
C1—N19—C20	113.6 (7)	C37—C39—H39A	109.5
C1—N19—H19A	108.8	C37—C39—H39B	109.5
C20—N19—H19A	108.9	H39A—C39—H39B	109.5
C1—N19—H19B	108.9	C37—C39—H39C	109.5
C20—N19—H19B	108.9	H39A—C39—H39C	109.5
H19A—N19—H19B	107.7	H39B—C39—H39C	109.5

### Hydrogen-bond geometry (Å, °)

**Table:**

*D*—H···*A*	*D*—H	H···*A*	*D*···*A*	*D*—H···*A*
O18—H18···O34	0.82	1.89	2.681 (10)	165
N19—H19A···O34^i^	0.90	1.75	2.568 (10)	149
N19—H19B···O18	0.90	2.46	2.772 (11)	101
N19—H19B···O22	0.90	2.29	2.646 (10)	103
O32—H32···O33^ii^	0.82	1.90	2.672 (10)	156
O35—H35···O36^iii^	0.82	1.99	2.619 (9)	132
O36—H36···O33	0.82	2.00	2.590 (9)	128

Symmetry codes: (i) −*x*, −*y*+1, −*z*+1; (ii) −*x*−1, −*y*+1, −*z*+1; (iii) −*x*−1, −*y*+1, −*z*.

## References

[bb1] Brook, C. S., Chen, W., Dell’Orco, P. C., Katrincic, L. M., Lovet, A. M., Oh, C. K., Spoors, P. G. & Werner, C. (2005). US Patent 20050240027A1.

[bb2] Chen, W.-M., Zeng, L.-M., Yu, K.-B. & Xu, J.-H. (1998). *Jiegou Huaxue*, **17**, 325–328.

[bb3] Chernyshev, V. V., Machula, A. A., Kukushkin, S. Y. & Velikodny, Y. A. (2009). *Acta Cryst.* E**65**, o2020–o2021. 10.1107/S1600536809029353 PMC2977435 21583690

[bb4] Dollase, W. A. (1986). *J. Appl. Cryst.* **19**, 267–272.

[bb5] Huber (2002). *G670 Imaging Plate Guinier Camera Software* Huber Diffraktionstechnik GmbH, Rimsting, Germany.

[bb6] Sheldrick, G. M. (2008). *Acta Cryst* A**64**, 112–122. 10.1107/S0108767307043930 18156677

[bb7] Spek, A. L. (2009). *Acta Cryst.* D**65**, 148–155. 10.1107/S090744490804362X PMC2631630 19171970

[bb8] Toraya, H. (1986). *J. Appl. Cryst.* **19**, 440–447.

[bb9] Werner, P.-E., Eriksson, L. & Westdahl, M. (1985). *J. Appl. Cryst.* **18**, 367–370.

[bb10] Yathirajan, H. S., Bindya, S., Sreevidya, T. V., Narayana, B. & Bolte, M. (2007). *Acta Cryst.* E**63**, o542–o544.

[bb11] Zhukov, S. G., Chernyshev, V. V., Babaev, E. V., Sonneveld, E. J. & Schenk, H. (2001). *Z. Kristallogr.* **216**, 5–9.

[bb12] Zlokazov, V. B. & Chernyshev, V. V. (1992). *J. Appl. Cryst.* **25**, 447–451.

